# PEERNaija—a mobile health platform incentivizing medication adherence among youth living with HIV in Nigeria: study protocol for a randomized controlled trial

**DOI:** 10.1186/s40814-023-01404-0

**Published:** 2023-10-27

**Authors:** Leslie J. Pierce, Martin C. Were, Sandra Amaral, Muktar H. Aliyu, Oliver Ezechi, Agatha David, Ifeoma Idigbe, Adesola Z. Musa, Prosper Okonkwo, Nadia Dowshen, Aima A. Ahonkhai

**Affiliations:** 1 Vanderbilt Institute for Global Health, Vanderbilt University Medical Center, Nashville, TN USA; 2 Department of Medicine, Vanderbilt University Medical Center, Nashville, TN USA; 3 Department of Biomedical Informatics, Vanderbilt University Medical Center, Nashville, TN USA; 4 Department of Pediatrics, Children’s Hospital of Philadelphia, Philadelphia, PA USA; 5 Center for Clinical Epidemiology and Biostatistics, University of Pennsylvania, Philadelphia, PA USA; 6 Department of Health Policy, Vanderbilt University Medical Center, Nashville, TN USA; 7 Nigerian Institute of Medical Research, Lagos, Nigeria; 8 APIN Public Health Initiatives (APIN), Abuja, Nigeria; 9 Craig-Dalsimer Division of Adolescent Medicine, Children’s Hospital of Philadelphia, Philadelphia, PA USA; 10 Perelman School of Medicine, University of Pennsylvania, Philadelphia, PA USA; 11 Department of Medicine, Infectious Diseases, Vanderbilt University Medical Center, Nashville, TN USA; 12 Massachusetts General Hospital, Division of Infectious Diseases, Boston, MA USA; 13 Harvard University Center for AIDS Research, Boston, MA USA

**Keywords:** Young adults, HIV, Adherence, Mobile health

## Abstract

**Background:**

Poor medication adherence is a major barrier to HIV control among youth living with HIV (Y-PLWH). The *PEERNaija* application (app) is an adapted smartphone app grounded in social cognitive and contigency management theories and designed to harness peer-based social incentives and conditional financial incentives to promote medication adherence. The app delivers a multifaceted medication adherence intervention including (1) peer-based social incentives, (2) financial incentives, (3) virtual peer social support, and (4) early clinic-based outreach for non-adherent Y-PLWH. A pilot trial of the app will be conducted in Nigeria, Africa’s most populous country with the 4th largest HIV epidemic, and home to 10% of the world’s four million Y-PLWH.

**Methods:**

In this randomized controlled trial, we will compare implementation outcomes (feasibility, acceptability, appropriateness measured via validated scales, enrollment and application installation rates, feedback surveys and focus group discussions with participants, and back-end application data), and preliminary efficacy (in improving medication adherence and viral suppression) of the *PEERNaija* app at 6 months. Participants in Arm 1 (*PEERNaija)* will receive daily medication reminders, peer-based social incentives, and virtual peer social support. Participants in Arm 2 (*PEERNaija* +*)* will additionally receive a conditional financial incentive based on their adherence performance. Eligibility for Y-PLWH includes (1) being aged 14–29 years, (2) being on ART, (3) owning a smartphone, (4) being willing to download an app, and (5) being able to read simple text in English.

**Discussion:**

This study will serve as the basis for a larger intervention trial evaluating the *PEERNaija* app (and the integration of mHealth, incentive, and peer-support-based strategies) to improve HIV outcomes in a critically important region of the world for Y-PLWH.

**Trial registration:**

ClinicalTrials.gov. NCT04930198. First submitted date: May 25, 2021. Study start: August 1, 2021, https://clinicaltrials.gov/. Protocol version: January 21, 2022.

## Introduction

### Background and rationale

The scale-up of global antiretroviral therapy (ART) represents an unparalleled global health success, leading to impressive overall reductions in HIV-related morbidity and mortality [1]. However, adolescents and young adults (AYA), especially those in Sub-Saharan Africa (SSA), have largely been left out of this progress. While AIDS-related deaths declined by 30% for adults from 2005 to 2012, AYA experienced a 50% increase in AIDS-related mortality over the same period [1–3]. Since then, survival among older AYAs has not substantially improved [4].

Unique developmental features of adolescence and young adulthood such as impulsivity, risk-taking, and present-focused, concrete thinking make chronic disease management particularly challenging in this population [5, 6]. In addition, AYA face psychosocial barriers to adherence and care, including fear of stigma and disclosure, leading them to often manage their disease in isolation [7–12]. As a result of these and other factors, AYA have poor medication adherence and higher rates of missed visits from HIV care than younger and older patients [13, 14]. The unfortunate impact of these behaviors includes unacceptably high rates of virologic failure (30–50%), virologic rebound after initial suppression (40%), and attrition from care (30–50%) in the year following ART initiation [5, 6, 13, 15]. Data from Nigeria and other settings show that medication adherence is a challenge even among those who consistently attend clinic visits [13]. One large multisite adherence study from Nigeria showed that one-quarter of AYA with optimal medication adherence, measured by pharmacy refill, still had virologic failure [14]. These data underscore the need for novel adherence interventions for this high-risk group.

Peer relationships provide an important and powerful premise for youth-focused interventions. In addition to being influenced by developmental factors, the attitudes and behaviors of young people are strongly influenced by their peers [16, 17]. Moreover, Bandura’s Social Cognitive Theory posits that behaviors and behavior change are informed by dynamic interactions between individuals and their environments and that health behaviors are guided by an individual's knowledge, self-efficacy, outcome expectations (cost and benefit of health habits), and health goals—all of which may be influenced by peers [18, 19]. While peer relationships often set the stage for poor health behaviors, a growing literature suggests that peer relationships can also be leveraged to promote the adoption and spread of positive health behaviors [20, 21]. Indeed, peer relationships have been increasingly utilized among youth (including African youth) to support outcomes along the entire HIV care continuum [22–24]. Given the central role of peer relationships for young people, conformity with peer behaviors can be re-envisioned as a unique type of social incentive for this age group [16, 17, 25]. Peer relationships may also impact knowledge, health goals, and outcome expectations (based on observed experiences of peers) of AYA.

Financial incentives are another approach to reinforcing or “nudging” patients toward desired outcomes. Financial incentives have been used in a variety of contexts to promote health behaviors for a range of medical conditions [26–28]. Based on the principles of contingency management theory, monetary incentives have been successfully utilized among AYA in SSA to prevent HIV infection, and among key adult populations to support ART adherence [29–31]. There are limited data, however, assessing incentives for ART adherence for AYA, especially in low-and middle-income countries (LMIC) [32]. Nonetheless, the provision of rewards for desired behaviors may be a particularly appealing approach for the adolescent brain which is primed to seek reward [33].

Digital health solutions, including text message reminders, have been added to the armamentarium of adherence support tools, and are recommended interventions for AYA living with HIV in some settings [34]. However, the full potential of mobile Health (mHealth) platforms to deliver novel behavioral interventions has not been realized. mHealth-based strategies are uniquely situated to deliver interventions via ecological momentary assessments—observing and intervening on health behaviors in real-time as people go about their daily lives, in real-world settings [35]. In addition, mHealth technologies can easily integrate novel gamification strategies which have been successfully used to address health behaviors by impacting intrinsic and extrinsic motivation [36, 37]. As such, there is potential for mHealth-based interventions to target a range of developmental and psychosocial barriers to medication adherence for AYA living with HIV.

### Choice of comparators

The multifaceted *PEERNaija* app incorporates medication reminders, peer-based social incentives, virtual peer support, and early outreach. Arm 1 will receive the *PEERNaija app* without financial incentive. Arm 2 will receive the *PEERNaija* app in addition to financial incentives. These comparator groups were chosen to allow us to evaluate implementation outcomes and to measure change from baseline adherence and viral suppression between (a) participants receiving the full range of app features (social support, early outreach, medication reminders) with embedded social incentives or (b) the full range of app features with social *and* financial incentives. While potentially additive in effect, it is unclear if and how the financial incentives will impact implementation and efficacy outcomes.

### Study objectives

The *PeerNaija* app is a smartphone application (app) grounded in social cognitive and contingency management theories. It was adapted from an app developed for youth living with HIV in the USA [37]. *PeerNaija* is designed to harness social incentives (from peer influence) and conditional financial incentives to promote medication adherence while providing virtual social support and early outreach to poorly adherent AYA living with HIV in an LMIC setting [36, 38]. The objective of this paper is to describe the protocol for a prospective pilot study of *PEERNaija*.

#### *Hypothesis*

We hypothesize that the *PEERNaija* app (with or without the financial incentive) will be a feasible, acceptable, and appropriate intervention for AYA living with HIV, and will exhibit high rates of adoption. Further, we hypothesize that the combination of social and financial incentives will show greater preliminary efficacy in improving ART adherence and HIV virologic control than the social incentive alone.

### Trial design

In this randomized controlled trial, we will compare the feasibility, acceptability, appropriateness, adoption, and preliminary efficacy of the *PEERNaija app* without financial incentive (arm 1) versus the *PEERNaija* app with financial incentive (*PEERNaija* + , arm 2) for ART adherence and HIV viral suppression among AYA living with HIV.

## Methods

### Study setting

The study will be conducted in Nigeria, Africa’s most populous country with the 4^th^ largest HIV epidemic globally [39, 40]. Nigeria is also home to approximately 10% of the world’s four million young (15–24 years old) people living with HIV (Y-PLWH) [41]. Participants will be recruited from the Nigerian Institute of Medical Research (NIMR). Located in Lagos, Nigeria, NIMR houses a comprehensive U.S. President’s Emergency Plan for AIDS Relief (PEPFAR)-funded HIV clinic with > 20,000 cumulatively enrolled adolescents and adults (≥ 15 years) and > 1000 children (< 15 years). NIMR also provides differentiated services for adolescents and young adults who receive their care during a specific adolescent clinic that meets monthly on weekends. This clinic is staffed by providers trained in adolescent medicine and incorporates enrichment activities, skill-building, and peer support. Preliminary data from NIMR confirmed high mobile phone ownership among Y-PLWH in NIMR (95% owned a mobile phone, 84% with smartphones) mirroring national data [41].

### Eligibility criteria

Patients will be eligible for this study if they:

1. Are 15–29 years of age
2. Own a smartphone (on which they are willing to download the *PEERNaija* app),
3. Are on ART, and
4. Demonstrate the ability to read simple text language in English.

### Intervention package

*PEERNaija* was developed as an Android-based app, and the theory-grounded development process is published elsewhere [36]. The Android platform was chosen to maximize feasibility as nearly 90% of mobile devices in Nigeria utilize this platform [42]. The *PEERNaija* app will feature daily medication reminders, with individual adherence monitoring, individual adherence scores based on daily monitoring, anonymized peer adherence scores (from peers attending the same clinic; social incentive), virtual peer support, and early clinic-based outreach for non-adherent youth. The comparator arm will receive this group of services along with a monthly lottery-based prize for youth with the highest adherence scores (financial incentive).

**Fig. 1:**
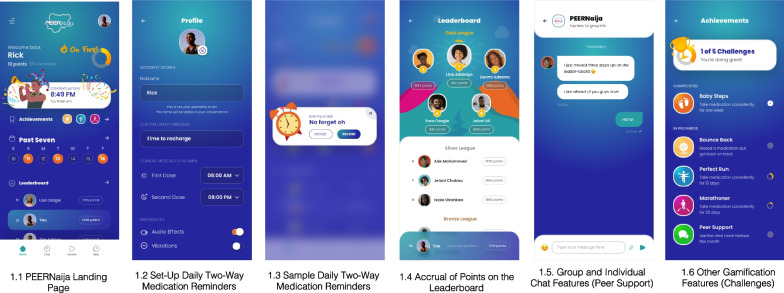
PEERNaija application features

Once consented, participants download the app onto their Android mobile device with the assistance of the study coordinator. To proceed with app setup, the participant must read and agree to the app's *Terms and Conditions, Privacy Notice*, and *Community Guidelines*. Subsequently, users can create an individualized profile, which includes (a) selecting an anonymous avatar and nickname, (b) choosing the dosing frequency of his/her ART medication regimen, (c) selecting preferred times for medication reminders, and (d) creating a preferred reminder message, or selecting from a pre-populated list. The multifaceted intervention includes app-based and non-app-based components described in detail below and shown in Fig. 1.

### PEERNaija application features

#### Daily two-way medication reminders

Participants receive daily medication reminders at the time(s) designated during setup. Medication reminder messages are programmed to first appear 30 min before the scheduled medication is due. Participants can either respond that the dose has been taken or ignore the reminder. If the reminder is ignored, it appears again at the time the medication is due, and every hour for up to 4 h which aligns with the clinic’s current HIV adherence counseling protocol. If the dose has not been recorded by the time the window period closes, the participant receives a message meant to encourage better adherence (Fig. 1 (1.2–1.3)). If the dose is taken and recorded within the 4-h window, the participant receives a message congratulating his/her adherence behaviors. This approach is informed by reports that two-way reminders are more effective than one-way medication reminders and that customized reminders are appealing to youth [43, 44]. Examples of these reminder messages are provided in Table 1.

**Table 1:** Sample reminder/response for two-way medication reminders

Reminder/response	Text
Daily medication reminder	“The time don reach”
	“Time to recharge”
	“How you dey?”
	“You don chop?”
All doses taken	“You finish am”
No doses taken	“You no get am well”
Some doses taken	“You can do beta”

#### Accrual of points on the leaderboard (social incentive)

Study participants accumulate 10 points daily if they take and record all medication doses within 4 h of the scheduled medication time. Based on individual cumulative point totals, participants are placed on a leaderboard and, as such, are keenly aware of their performance relative to their peers via their anonymous avatars. In addition, the app tracks individual adherence scores (percentage of prescribed doses taken), and participants are displayed on the leaderboard according to an adherence tier (Fig. 1 (1.1, 1.4)). We established three tiers—gold, silver, and bronze—based on historical thresholds of medication adherence that correlate with virologic suppression [33, 45, 46]. Optimal adherence is defined as adherence of > 94% and puts participants in the gold league, suboptimal adherence is defined as adherence between 80 and 94% (“silver league”), and poor adherence is defined as adherence < 80% (“bronze league”). Points and adherence scores are reset at the beginning of each month to give opportunities for new users or users with previous poor scores a chance to receive the highest scores.

#### Normative messaging (social incentive)

One way in which peer relationships may influence behaviors is through the exertion of descriptive norms (what is most commonly done or what individuals perceive to be so) and injunctive norms (what ought to be done) for certain behaviors [35, 47]. In the *PEERNaija* app, the display of the individual’s adherence score relative to peer scores is considered a *descriptive norm* and portrays how most peers are doing with regard to adherence. Participants will also receive an *injunctive norm*, or an indication of what they ought to be doing. When coupled with descriptive norms, injunctive norms have counteracted regression to the mean for individuals who demonstrate desirable behaviors relative to their peers [35, 47]. Informed by this theoretical background, our team developed culturally and age-appropriate normative messages for participants in each adherence tier. These messages will begin to appear the second week of each month so that individuals have a chance to establish adherence behaviors. Sample messages are shown in Table 2.

**Table 2:** Sample normative messages based on adherence tier relative to peers

Optimal adherence (> = 95%)	Suboptimal adherence (< 95%)	Suboptimal adherence (< 95%; > 80%) but higher than peers	Poor adherence (< 80%) but higher than peers
[Username], you don try well well! You don reach [user adherence rate]	Well done o [Username]. Your paddy dem for PEERNaija don reach [group adherence rate] but you dey [adherence rate]. No lose focus o/ No lose guard o	[Username], you are leading the pack! You can still do better!	[Username], you don reach [adherence rate]. No lose guard o
Dear [Username], you too much! You don reach [user adherence rate]	Well done [Username]. You dey [adherence rate]. Your paddies for PEERNaija don reach [group adherence rate]. Do quick catch dem!	Ah, [Username] you dey lead oh, no lose guard!!!	[Username], you don reach [adherence rate]. No lose focus o
[Username], you don reach [user adherence rate]. Well done o!	Hey [Username], you dey [adherence rate]. Others for PEERNaija don reach [group adherence rate]. Make you try reach am!	[Username] ride on!	[Username], you can still do better!
Hey [Username], your score don reach [user adherence rate]. Maintain the vibe!	Dear [Username], you dey do well well! You don reach [adherence rate]. Your paddies on PEERNaija dey [group adherence rate] already	You dey try, keep riding!!!	[Username], you don reach [adherence rate]. No lose guard o
Well done [Username]. You bin reach [user adherence rate]. Na small e remain	[Username], your score don reach [user adherence rate]%. E remain small!	Keep grinding [Username]	
Well done [Username], you done finish [user adherence rate]. You almost chop am finish. Continue!	Hey [Username], [percentage of users in optimal adherence] just got 95%, level up!	[Username] push na	

#### Group and individual chat features (peer support)

*PEERNaija* includes both individual and group-based chat features to allow users to virtually communicate, and provide peer camaraderie and support in a safe, anonymous setting. Participants are required to consent to follow community rules for chatting (Fig. 1 (1.5)). Participants who have not participated in the group chat for 7 days receive a notification stating “We’ve missed you!” to encourage chat participation and engagement.

#### Help tab

Participants will be able to reach out directly and individually to the research assistant through the help tab on the landing page which will be linked to the chat feature on the *PEERNaija* Provider App. This help button is not for medical emergencies, and the participants will be counselled extensively about this. The Research Assistant will be responsible for monitoring these chats on a daily basis. The Research Assistant will be required to follow up with the appropriate personnel for the request. Examples include but are not limited to clinic appointment requests (in which an appointment is scheduled within 24 h), health-related questions (forwarded to the HIV Clinic Director at the end of each day and a response is provided to the participant within 48 h), app-specific questions (addressed by the Research Assistant or on-site IT Assistant), or app errors (on-site IT Assistant is engaged and the US-based program manager and app developers are informed as needed).

#### Leaderboard and lottery (financial incentive)

For the financial incentive**,** the top 5 scorers on the leaderboard in the *PEERNaija* + study arm will be eligible to win a lottery prize each month of the 24-week pilot. The winner will be randomly drawn, and the study coordinator will remotely load 1000 NGN (approximately 1.5 GB) of “data” directly onto the winner’s phone. The winner will also be announced in the group chat.

#### Other gamification features

Specific achievements can be unlocked based on the number of days of perfect adherence and are illustrated in Fig. 1 (1.6). By their very nature, adherence-related achievements become unlocked in a stepwise fashion. When an achievement is unlocked, the participant receives a badge and a congratulatory message. These achievements reset every month. In response to user feedback, we have also added a page that allows participants to download popular app-based games such as Candy Crush, Temple Run, and Ludo King onto their devices.

#### Provider app and escalated outreach

A *PEERNaija* Provider app was developed to allow the study team to monitor participant’s self reported medication adherence in real time. The provider app features an activity tab, leaderboard, and chat feature. The activity summarizes each participant’s study identification number, the most recent time medication was recorded, the most recent time the group chat feature was used, and the most recent time the participant app was opened. The leaderboard on the provider app mirrors the leaderboard in the participant app and includes each participant’s accrued points, and a symbol signifying either no, negative, or positive change in the user’s rolling 7-day adherence. With this simple presentation, participants whose adherence has decreased over a 7-day period can easily be identified for outreach. In this way, adherence challenges can be addressed in real time rather than waiting until they are discovered during clinic visits or laboratory assessments.

Outreach will occur in a stepwise fashion. The NIMR clinic has a standard outreach protocol for patients who have missed clinic visits or are struggling with medication adherence. Phone calls are the first line of action. If the participant is reached, a member of the outreach team will inquire about the participant’s current health status (i.e. ask how they are doing, if they are on medication and adhering to medication). The outreach member will explore reasons why the participant’s recorded adherence has declined (recognizing that this could be related to application logistics, logging behavior, or medication-taking behavior), and will either troubleshoot logistical challenges or conduct a brief counseling intervention to reinforce the importance of adherence, as appropriate. Alternatively, if the counselor is unable to get in touch with the participant, the counselor will reach out to the next of kin as provided by the participant. If the attempts to call the participant and/or next of kin were unsuccessful, the Research Assistant will schedule a home visit. Either the Research Assistant or an identified member of the home-based care team will be assigned to the home visit. Home visit activities will include counseling as described above.

### Primary outcomes

Primary outcomes include feasibility, acceptability, and adoption of the *PEERNaija* intervention measured at the end-user level.

### Secondary outcomes

The secondary outcome for our study is preliminary efficacy which will be assessed by comparing change in HIV viral load and adherence from baseline to 24 weeks in the two study arms.

### Study arms

There will be 2 study arms [*PEERNaija and PEERNaija* +].

#### Arm 1

Arm 1 of *PEERNaija* receives all of the intervention components (two-way medication reminders, leaderboard, normative messaging, peer chat, challenge features, and escalated outreach for poor adherence).

#### Arm 2

Arm 2, or *PEERNaija* + *,* receives all of the intervention components in the *PEERNaija* arm. In addition, participants in this arm are eligible for the financial incentive which is awarded randomly to a participant in the top 5 on the leaderboard at the end of each study month.

### Recruitment process

Participants will be recruited (1) via fliers prominently displayed in the clinic, (2) by referral from their clinical providers and counselors during routine and adolescent clinic visits, (3) during health talks (which occur in the waiting area on clinic days while patients are waiting to be seen by their providers), and (4) via direct telephone outreach from study coordinators. Patients who decide to enroll in the study will provide written informed consent. Those who decline to participate will be asked to complete a brief questionnaire detailing their reasons for doing so. As per Nigerian ethics guidelines, “persons aged 16 and above and emancipated minors can consent for themselves without parental consent”. Both parental consent and assent from the minor are required for persons aged 10–15 [48]. Patients, and a parent/guardian, where required, will provide written informed consent for study participation.

### Sample size and randomization

A total of 50 participants will be randomized 1:1 to receive either *PEERNaija* or *PEERNaija* + . Because of the nature of this intervention, neither participants nor study staff will be blinded to randomization status. Our sample size of 50 was primarily chosen based on logistical feasibility for this single-site pilot study considering new client enrollment, virologic failure rate in the first year on ART, SMART phone ownership, and loss-to-follow-up. This information will inform sample size calculations for a larger trial based on this pilot data.

### Ethical approval

All subjects are required to provide their informed consent for inclusion before participation in the study. The protocol was approved by the Ethics Committee of Vanderbilt University Medical Center (#200,116), the Nigerian Institute of Medical Research (#20/005), and APIN Public Health Initiatives (IRB024-FR).

### Study measures

#### Data collection tools and procedures

Participants will be seen at the HIV clinic at enrollment, 12 weeks, and 24 weeks (Table 3). At enrollment, participants will complete a *demographic* assessment. At baseline and all follow-up visits, participants will complete a comprehensive psychosocial survey assessing depression (PHQ-9), ART self-efficacy (medication-taking self-efficacy scale), HIV stigma (Berger’s HIV stigma scale), and social support (SPS-10 scale) [49–52].

**Table 3:** Schematic of study measures

	Study period		
Timepoint	Enrollment	Post-randomization (12 weeks)	Close out
Enrollment			
Eligibility screen	X		
Informed consent	X		
Randomization	X		
Intervention			
PeerNaija	X	X	
PeerNaija +	X	X	
Assessment			
App orientation	X		
Demographics survey	X		
Psychological survey	X	X	X
*Depression (PHQ-9)	X	X	X
ART Self-Efficacy (MT SES Scale)	X	X	X
HIV Stigma (Berger's Scale)	X	X	X
Social Support (SPS-10)	X	X	X
Alcohol and Substance Use (CRAFFT)	X	X	X
Adherence assessment	X	X	X
Visual analogue scale	X	X	X
Medication possession ratio	X	X	X
ACTG questionnaire	X	X	X
App usage statistics		X	X
Clinical measures	X		X
CD4 cell count	X		X
HIV RNA	X		X
Implementation outcomes		X	X
Feasibility, acceptability, appropriateness surveys		X	X
Feedback survey		X	X

### *Implementation outcomes* include feasibility, acceptability, and adoption at 12 and 24 weeks

Feasibility will be assessed by documenting the ability to enroll participants (enrollment rate), and successfully install the application (installation rate). Operational feasibility will be evaluated by summarizing the server function and stability (number of server outages that impacted application functionality), monitoring the successful delivery of medication reminders (back-end data analysis), data availability, usage, and security issues (participant feedback surveys). Participants will also report on potential structural challenges, such as the strength and availability of wireless signals and the degree of device sharing (participant feedback surveys). Weiner’s feasibility of the intervention, acceptability of the intervention, and intervention appropriateness measures (FIM, AIM, IAM) will be administered to study participants to assess feasibility and acceptability appropriateness. These are each 4-item tools scored on a 5-point scale (1–5, 5 is the most positive score) [53]. Through feedback surveys, participants will also provide tailored feedback on the application features, including the likelihood of using the application, value added by the application, most and least desired features, and impact on medication-taking behavior. Feasibility, acceptability, and appropriateness of the intervention will also be assessed via 2 focus group discussions, comprised of 5 to 7 participants in each group conducted after a 24-week follow-up has been completed. Adoption of the adherence reminder features will be measured by a priori codes established for task engagement, completion, and efficiency. Task engagement is the primary adoption measure of interest, and will be defined as the time spent logged onto the app, and using each app feature. We will measure task engagement using time stamps, allowing us to calculate the time the user logged onto and off the app, and navigate to the adherence reminder feature. Task completion will be defined as the presence or absence of a response to time-based adherence reminders. We will measure the proportion of “completed,” and “ignored” responses to reminders for each participant. Task efficiency will be defined as the time it takes for the participant to respond to the adherence reminder. Finally, chat feature use and content will be measured by monitoring the monthly frequency of posts for each participant in addition to the thematic content. The content will be accessed from a study coordinator application that will allow selected team members to monitor the chat posts and download them for content analysis. While we will assess these findings in aggregate to determine overall study feasibility, we have also identified a priori feasibility thresholds including the ability to achieve recruitment targets, retain > 80% of participants in follow-up (without discontinuation due to device, application, or IT infrastructure challenges), and an average score of 70% or greater on FIM, AIM, and IAM surveys.

Clinical measures will include ART adherence, CD4 + cell count, and HIV RNA. Adherence will be measured at baseline, 12, and 24 weeks and will be measured in the following ways: percentage of doses documented in the PEERNaija app, self-reported adherence using the visual analog scale, medication possession ratio from pharmacy records (refilled doses/prescribed doses at the central pharmacy for the clinic utilized by all participants), and AIDS Clinical Trials Group adherence questionnaire [45, 54, 55]. Baseline HIV RNA and CD4 + count will be assessed within 3 months of study enrollment, and again within 4 weeks of study completion.

### Data and outcome analysis

Study feasibility, acceptability, appropriateness, and adoption are the primary outcomes for this pilot trial and will be determined by analyzing the content of the feedback surveys at each study visit, and focus group discussions at the study end. Additionally, we will summarize the average Weiner’s FIM, AIM, scores, document recruitment, application installation, and the proportion of medication reminders that are successfully delivered to participants. Adoption will additionally be assessed by analyzing usage data as described above.

Preliminary efficacy will be assessed as a secondary outcome by conducting a pre–post-comparison of adherence measures, HIV viral load, and adoption measures at baseline, 12, and 24 weeks in the two study groups (*PEERNaija and PEERNaija* +*).* Descriptive and distributional analyses will be performed to describe the sample at baseline and during follow-up assessments. The study will not be powered to detect statistically significant differences in adherence measures or viral load, either within or between intervention groups, but will inform sample size considerations for future randomized interventions. However, preliminary efficacy will be assessed by conducting a pre–post-comparison of adherence measures, HIV viral load, and adoption measures at baseline, 12, and 24 weeks in the two study groups (*PEERNaija and PEERNaija* +*),* using paired tests (e.g., paired *t*-tests, signed rank tests, or McNemar’s tests, as appropriate), with a significance level set at *p* ≤ 0.05 (2-tailed test). Multivariable analyses (e.g., logistic regression and cumulative probability models) will also be performed, adjusting for baseline measures and potential confounders such as sex, (younger 15–19 years vs older 20–24 years age), baseline depression (SRQ-20 scale), baseline symptom score (linear), baseline social support (categorical, low vs. high), and baseline CD4 + count (categorical) identify factors associated with rates of application adoption. We will also assess whether the addition of financial incentives (*PEERNaija vs, PEERNaija* +) is associated with higher rates of app adoption at each follow-up time point. Focus group discussions will be recorded, and framework analysis will be used to identify main themes about satisfaction with the app, facilitators and barriers to adoption, and perceived impact on adherence behaviors. Finally, to assess information obtained from feedback surveys, usage data responses rating specific application features will be compared to application adoption measures using Kruskall-Wallis tests.

### Quality control mechanisms

The quality of the study outputs will be ensured by employing validated data collection tools. In addition to the creation and implementation of operations manuals, data collectors and supervisors will be trained on all data collection tools and procedures. One Research Assistant will manage the mobile app technology to ensure proper functioning, two Nigerian-based research assistants will coordinate the overall research, and one US-based research assistant will provide continuous supervision throughout the entire follow-up.

Recognizing that loss of privacy is a serious issue, especially for an mHealth-grounded intervention for youth, measures will be put in place to protect identifiable data [56]. First, all investigators and research personnel will receive training on the protection of human subjects. Second, identifiable information will only be made available to a few specific research personnel who will need access to the data purely for study purposes. All paper records will be stored in secure locked cabinets only accessible to study personnel. Third, the mHealth application was developed with security as the top priority. The security features include storage of data in an encrypted format within a password-protected smartphone, secure https-based data transmission between the mHealth application and the server based in Nigeria, robust user-level authentication to access data, and automatic log-out after a period of inactivity. Other features that minimize risk to confidentiality and privacy include utilizing neutral signifiers on the application, that would not be readily linked to HIV status (for example using the text “it’s time” or another chosen text to pop up on the phone screen when it is time to take an ART dose, not including any symbols or logos readily linked to HIV, and not using the name of the clinic or the participants on the application screen). The choice of text and formatting was informed by user-centered design. Fourth, participants will provide specific consent for the provision of phone or home-based outreach if the mHealth app signals non-adherence for 5 days or more. Concerted efforts will be made to minimize the use of any paraphernalia (labeled vehicles, clothing on clinic staff) that might identify participants as HIV patients, and no communication will be initiated in the presence of individuals who are not authorized by the participant to be present. Nonetheless, this risk will be explicitly discussed with participants at the time of enrollment and consent for study participation. Fifth, participants will be counseled on the low risk of data breach with these safeguards, but also on how to further minimize data breach when phones are shared. These safeguards will be present in the demonstration session introducing the participants to the application.

### Ethics and dissemination

Adverse events that occur after participants consent to the study including unanticipated disclosure will be monitored by the study team and reported to the VUMC and AIDS Prevention Initiative (Nigerian IRB body) IRBs within 72 h. The findings from this proposal will be shared as abstracts at international HIV conferences, and submitted for publication in peer-reviewed journals.

### Patient and public involvement plan

End users and stakeholders in the field of HIV have been a critical component of the PEERNaija development process. Their involvement will continue to help shape the mobile application content and the timing and frequency of medication adherence reminders. In addition, throughout the data collection and follow-up period, the end users and stakeholders will provide key perspectives on the challenges of implementing health-based mobile applications among Nigerian AYA.

## Conclusion

This study will provide important data for the potential role of smartphones in delivering incentive-based medication adherence interventions along with essential peer support for vulnerable youth living with HIV. In addition, if proven to be effective, the inclusion of financial incentives will add additional costs that could impact scalability. The project will serve as the basis for a larger intervention trial evaluating this mHealth application in a critically important region of the world for AYA living with HIV, while building capacity for a large programmatic network to conduct future mHealth research in this setting. The intervention has the potential to improve medication adherence, and therefore virological control and survival in a very high-risk patient population.

## Data Availability

Data sharing is not applicable.
